# Multicentre sleep‐stage scoring agreement in the Sleep Revolution project

**DOI:** 10.1111/jsr.13956

**Published:** 2023-06-13

**Authors:** Sami Nikkonen, Pranavan Somaskandhan, Henri Korkalainen, Samu Kainulainen, Philip I. Terrill, Heidur Gretarsdottir, Sigridur Sigurdardottir, Kristin Anna Olafsdottir, Anna Sigridur Islind, María Óskarsdóttir, Erna Sif Arnardóttir, Timo Leppänen

**Affiliations:** ^1^ Department of Technical Physics University of Eastern Finland Kuopio Finland; ^2^ Diagnostic Imaging Center Kuopio University Hospital Kuopio Finland; ^3^ School of Information Technology and Electrical Engineering The University of Queensland Brisbane Queensland Australia; ^4^ Reykjavik University Sleep Institute, School of Technology Reykjavik University Reykjavik Iceland; ^5^ Department of Computer Science Reykjavík University Reykajvík Iceland

**Keywords:** agreement, multicentre, scoring, sleep, sleep stage

## Abstract

Determining sleep stages accurately is an important part of the diagnostic process for numerous sleep disorders. However, as the sleep stage scoring is done manually following visual scoring rules there can be considerable variation in the sleep staging between different scorers. Thus, this study aimed to comprehensively evaluate the inter‐rater agreement in sleep staging. A total of 50 polysomnography recordings were manually scored by 10 independent scorers from seven different sleep centres. We used the 10 scorings to calculate a majority score by taking the sleep stage that was the most scored stage for each epoch. The overall agreement for sleep staging was *κ* = 0.71 and the mean agreement with the majority score was 0.86. The scorers were in perfect agreement in 48% of all scored epochs. The agreement was highest in rapid eye movement sleep (*κ* = 0.86) and lowest in N1 sleep (*κ* = 0.41). The agreement with the majority scoring varied between the scorers from 81% to 91%, with large variations between the scorers in sleep stage‐specific agreements. Scorers from the same sleep centres had the highest pairwise agreements at *κ* = 0.79, *κ* = 0.85, and *κ* = 0.78, while the lowest pairwise agreement between the scorers was *κ* = 0.58. We also found a moderate negative correlation between sleep staging agreement and the apnea–hypopnea index, as well as the rate of sleep stage transitions. In conclusion, although the overall agreement was high, several areas of low agreement were also found, mainly between non‐rapid eye movement stages.

## 1 INTRODUCTION

Accurate identification of sleep stages comprises the cornerstone of diagnostic sleep medicine, as well as sleep research (Berry et al., 2020; Rechtschaffen & Kales, 1968). Sleep stages are scored by visually evaluating typical sleep stage‐related patterns and features in electroencephalography (EEG), electro‐oculography, and electromyography. The recorded signals are divided into 30‐s epochs and a single sleep stage is identified for each epoch. The features used for sleep staging include both the frequency characteristics of the signals, as well as distinct features such as sleep spindles, slow‐wave oscillations, and ‘saw‐tooth’ waves. These allow distinguishing rapid eye movement (REM) sleep, three stages of non‐REM (NREM) sleep (N1, N2, and N3), and wakefulness during the night. Finally, the sleep staging from the whole night results in a hypnogram used to evaluate both the sleep cycles and the quality of sleep, and assessment of important sleep indices such as total sleep time, sleep onset latency, and time spent awake after sleep onset (Berry et al., 2020).

The current sleep stage scoring rules have been revised from the original Rechtschaffen and Kales standard (Rechtschaffen & Kales, 1968) and are now based on rules formulated by the American Academy of Sleep Medicine (AASM; Berry et al., 2020). Despite the common sleep stage scoring rules (Berry et al., 2020; Rechtschaffen & Kales, 1968), there still exists considerable variation in the scoring between different scorers (Danker‐Hopfe et al., 2009; Magalang et al., 2013; Norman et al., 2000; Penzel et al., 2013; Rosenberg & Van Hout, 2013; Zhang et al., 2015). Significant variations exist especially between centres each interpreting the rules slightly differently with distinct protocols and practises. These differences exist even in the healthy population and become even more apparent in patients with sleep‐disordered breathing (Norman et al., 2000; Penzel et al., 2013), most likely due to fragmented sleep with abnormal sleep patterns and frequent sleep stage transitions. Overall, the transition from the Rechtschaffen and Kales scoring rules to the AASM scoring rules has provided improvements in the scoring agreement (Danker‐Hopfe et al., 2009), but the inter‐scorer agreement could still be improved significantly. For these reasons, investigating the agreement in sleep staging practises systematically in a multicentre and multidisciplinary manner is crucial to gaining a better understanding of the shortcomings of current sleep staging rules and identifying the uncertain areas with the most significant variation in scoring.

There have been previous efforts investigating sleep staging agreement (Bakker et al., 2022; Danker‐Hopfe et al., 2009; Magalang et al., 2013; Norman et al., 2000; Penzel et al., 2013; Rosenberg & Van Hout, 2013; Younes et al., 2016; Zhang et al., 2015). These have reported inter‐rater agreements measured by kappa coefficients of *κ* = 0.63 (Magalang et al., 2013) to *κ* = 0.76 (Danker‐Hopfe et al., 2009) for the sleep staging within the multicentre Sleep Apnea Genetics International Consortium (SAGIC; Magalang et al., 2013) and SIESTA (Danker‐Hopfe et al., 2009) datasets, respectively. The individual sleep stage accuracies have varied from *κ* = 0.31 (Magalang et al., 2013) to *κ* = 0.46 (Danker‐Hopfe et al., 2009) for N1 sleep, and from *κ* = 0.78 (Magalang et al., 2013) to *κ* = 0.91 (Danker‐Hopfe et al., 2009) for REM sleep. These studies utilised 15–72 polysomnography (PSG) recordings and relied on two to nine scorings for each recording (Danker‐Hopfe et al., 2009; Magalang et al., 2013; Younes et al., 2016). Similarly, the AASM inter‐scorer reliability programme has reported an overall agreement of 82.6% when using nine record segments and >2000 scorers (Rosenberg & Van Hout, 2013). Bakker et al. (2022) have also recently studied sleep staging agreement in three datasets with a varying number of recordings (10–70) and scorers (six to 12), but focused mostly on automatic scoring with limited metrics on manual sleep stage agreement. However, most of the previous studies have had a low number of scorers or recordings or have only used short recording segments instead of the whole night. In addition, the studies have only reported quantitative sleep parameters or overall agreement metrics without providing more detailed investigations of the segments with disagreement in scoring.

Therefore, the aim of this study was to provide a comprehensive and detailed evaluation of the inter‐rater agreement in sleep staging. Our aim was to focus on highlighting the areas of disagreement and the possible reasons behind these disagreements instead of only reporting the agreement values. The sleep staging agreement was evaluated between specialised sleep units across Europe and Australia within the Sleep Revolution project (Arnardottir et al., 2022). We utilised 50 PSG recordings, each scored once by 10 experienced individual scorers from seven different sleep centres to investigate the agreement and highlight the uncertain areas with the most disagreement in scoring. We hypothesised that the agreement in scoring N1 sleep would remain the lowest, as reported in previous studies, and that the transitions between sleep stages would cause uncertainty in the scoring.

## 2 METHODS

### 2.1 Dataset

This study was based on 50 prospective type II PSG recordings conducted at Reykjavik University from February 2021 to June 2021 using a Nox A1 device (Nox Medical, Reykjavik, Iceland). To get a well‐rounded study population, the subjects were recruited based on information gathered from online screening questionnaires. The initial goal was to have a similar ratio of subjects from the following groups: obstructive sleep apnea (OSA) risk group, restless leg syndrome (RLS) risk group, insomnia risk group, and healthy individuals. However, this goal was not fully reached in the end and the population ended up slightly OSA risk dominant. The STOP‐BANG (snoring, tiredness, observed apnea, high blood pressure, body mass index, age, neck circumference, and male gender) questionnaire (Chung et al., 2016) was used to assess OSA risk, the Insomnia Severity Index (Morin et al., 2011) was used to assess insomnia risk and the International Restless Legs Syndrome Study Group Questionnaire (Horiguchi et al., 2003) was used for determining RLS risk. The RLS risk was determined in the same manner as in Benediktsdottir et al. (2010). The study was approved by National Bioethics Committee of Iceland (21–070, 16.3.2021). All subjects gave written informed consent to participate in the study.

All 50 PSGs were manually scored by 10 scorers from seven different sleep centres (Table 1) between April 2021 and September 2021. All scorers used Noxturnal software, Research Version 6.1.0.30257 (Nox Medical, Reykjavik, Iceland) for scoring. The scorers were instructed to follow the AASM scoring rules, version 2.6 (Berry et al., 2020) with no deviations. Recommended rules were always used instead of the alternative rules. The same workspace template was shared with all scorers, which could be modified according to each scorer's preferences. Noxturnal software does not highlight spindles or delta waves, but axis lines could be applied to view the 75 μV threshold. Each scorer also separately checked the recording quality and marked sections with signal loss or insufficient signal quality for accurate scoring as invalid periods and no scoring was performed during these periods. The scorers were instructed to score in the following order: sleep stages and arousals, respiratory events and desaturations, leg movements, and the classification of arousals. In this paper, we have only focused on sleep stage scoring. The subjects’ characteristics data are presented in Table 2. The completed scorings for all scorers and subjects were exported (Nikkonen et al., 2022a, b) as xls‐files and all data analyses were performed using Matlab R2022a.

**TABLE 1:** Participating sleep centres.

Sleep centre	Participating scorers
Charite–Universitaetsmedizin Berlin, Berlin, Germany	2
Istituti Clinici Scientifici Maugeri Spa Società Benefit, Pavia, Italy	1
Princess Alexandra Hospital, Brisbane, Australia	1
Reykjavik University, Reykjavik, Iceland	2
Turku University Central Hospital, Turku, Finland	1
University of Gothenburg, Gothenburg, Sweden	1
University of Lisbon, Lisbon, Portugal	2

**TABLE 2:** Subjects’ characteristics data.

	N(%)
Total number of subjects	50 (100)
Male subjects	29 (58)
Female subjects	21 (42)
Subjects with obstructive sleep apnea risk	29 (58)
Subjects with insomnia risk	17^a^ (36)
Subjects with restless legs syndrome risk	11 (22)
No‐risk subjects	9^a^ (19)
	**Mean(SD)**
Age, years	42.9 (13.7)
Body mass index, kg/m^2^	27.3 (5.8)
Total sleep time, min	426.3 (57.9)
Apnea–hypopnea index, events/h	15.2 (15.6)
Arousal index, events/h	18.4 (7.2)
Oxygen desaturation index, events/h	14.5 (16.3)
Wake after sleep onset, min	1 (1)
Sleep efficiency^b^	1 (1)
Epworth Sleepiness Scale score	9.2 (4.8)

^a^ Insomnia risk data was missing from three subjects. Subjects were defined as no‐risk if they had no obstructive sleep apnea, restless legs syndrome or insomnia risk.

^b^ Mean sleep efficiency calculated from the analysed period instead of time in bed.

### 2.2 Data analysis

To aid in evaluating the scoring agreement, we used the 10 manual scorings to calculate a majority score for each analysed epoch. The majority score was formed by taking the sleep stage that was the most scored stage for that epoch. For example, if two scorers had scored the stage as N2, five scorers the stage as wake, and three scorers the stage as N1, the majority score was wake. If there was a tie between two or more stages, the stage higher in the tiebreaker order was chosen. The arbitrarily chosen tiebreaker order was: wake, N1, N2, N3, REM.

As some of the recordings included excessive amounts of scored wake before and after the sleep period, we defined the analysis period to start from the first non‐wake epoch scored by any scorer and to end at the last non‐wake epoch scored by any scorer. Thus, effectively all excess wake periods were trimmed from the start and end of the recordings. In addition, as different scorers started and ended their analyses at different times, the unscored epochs before the first scored epoch and after the last scored epoch were considered as wake. This trimming method also makes the amount of scored wake directly comparable between the scorers as the exact same epochs are considered for each scorer.

We evaluated the scoring agreement using *κ* statistics as well as calculating the observed agreement and agreement with majority scoring metrics. We used Cohen's *κ* (*κ*_c_; Cohen, 1960) for pairwise comparisons and Fleiss’ *κ* (*κ*_f_; Fleiss, 1971) for multi‐rater comparisons. To get sleep stage‐specific *κ* values for comparison with previous studies, the *κ*_f_ for each sleep stage was also calculated in a binary manner, that is, each sleep stage was individually compared to all other stages. Thus, this binary *κ* (κ_fb_) represents more of a detection accuracy for each sleep stage. However, it should be noted that as the *κ* values are dependent on the number of scorers and the number and length of recordings, the *κ* values between different datasets cannot be directly compared. In addition, we calculated the agreement in each sleep stage based on the majority score in the standard five sleep stages and additionally when N1 and N2 were combined into a light sleep stage. We also calculated how the number of sleep stage transitions and respiratory events affect the scoring agreement. For this analysis, we calculated confusions between sleep stages. A confusion expressed an epoch that had mixed scoring of two stages from scorers that is, an N1/N2 confusion would be an epoch where, e.g., two scorers had scored the epoch as N1 and eight scorers as N2. The level of agreement did not matter for this calculation, that is, 3 × N1 + 7 × N2 was counted similarly as an N1/N2 confusion as 6 × N1 + 4 × N2. Finally, we calculated the proportions of all the different scoring combinations between the five sleep stages and confusion matrices for each scorer against the majority scoring.

## 3 RESULTS

The overall agreement across all sleep stages was *κ*_f_ = 0.714. The agreement was highest when the majority sleep stage was REM and lowest when the majority sleep stage was N1 (Table 3). The agreement was also calculated for N1 and N2 as a combined light sleep stage (Table 3). The agreement remained highest when the majority sleep stage was REM, and the mean agreement with the majority score was lowest when the majority sleep stage was the light sleep stage. However, the observed agreement of light sleep stage was then higher than for wake and N3. The κ_fb_ values were 0.763 for wake, 0.406 for N1 sleep, 0.673, for N2 sleep, 0.696 for light sleep, 0.742 for N3 sleep, and 0.861 for REM sleep.

**TABLE 3:** Scoring agreement in each sleep stage across all 50 subjects.

Standard five stages			N1 and N2 combined to light sleep		
Majority sleep stage	Mean agreement with majority scoring, %	Observed agreement, %	Majority sleep stage	Mean agreement with majority scoring, %	Observed agreement, %
All sleep stages	86.3	79.1	All sleep stages	89.5	83.7
Wake	86.3	79.3	Wake	87.6	81.4
N1	65.6	51.3	Light sleep	82.7	83.2
N2	86.0	78.8			
N3	88.8	82.0	N3	89.0	82.2
REM	91.7	86.5	REM	92.6	87.9

Abbreviation: REM, rapid eye movement.

*Note*: observed agreement was defined as the number of rater pairs in agreement relative to the number of all possible rater pairs that is, the observed agreement without being adjusted by chance agreement.

The total minutes scored of each sleep stage varied between the scorers (Table 4). The variation was greatest in N1 where, e.g., scorer 8 scored over three times the N1 compared to scorer 3. The variation was lowest in REM where even the largest difference between the scorers was <25%.

**TABLE 4:** Total scored minutes of each sleep stage across all 50 subjects.

Variable	Wake	N1	N2	N3	REM	TST
Majority score, min	2985.5	1648.0	10,544.0	4449.5	4651.0	21,292.5
Proportion of all epochs, %	12.3	6.8	43.4	18.3	19.2	87.7
Scorer 1, min	3328.0	2635.0	8216.5	5316.0	4782.5	20,950.0
Scorer 2, min	3340.5	2071.5	10,239.0	3744.5	4882.5	20,937.5
Scorer 3, min	2513.0	987.0	11,605.0	4675.5	4497.5	21,765.0
Scorer 4, min	2657.5	2096.5	10,278.5	5075.0	4170.5	21,620.5
Scorer 5, min	3136.5	1771.5	9497.5	5259.5	4613.0	21,141.5
Scorer 6, min	2870.5	1205.5	11,282.0	4336.5	4583.5	21,407.5
Scorer 7, min	2878.0	1498.0	10,773.5	4428.0	4700.5	21,400.0
Scorer 8, min	1942.5	3023.0	9337.5	5457.0	4518.0	22,335.5
Scorer 9, min	3183.0	1785.0	9015.0	6373.0	3922.0	21,095.0
Scorer 10, min	3782.0	1849.0	11,228.0	2658.5.0	4760.5	20,496.0
Mean disagreement with majority scoring, min	390.9	494.9	939.2	809.0	212.3	390.9
Mean disagreement with majority scoring, %	13.1	30.0	8.9	18.2	4.6	1.8

Abbreviations: REM, rapid eye movement; TST, total sleep time.

*Note*: Excess wake was trimmed from before and after sleep and thus the amount of wake is directly comparable between the scorers.

**FIGURE 1:**
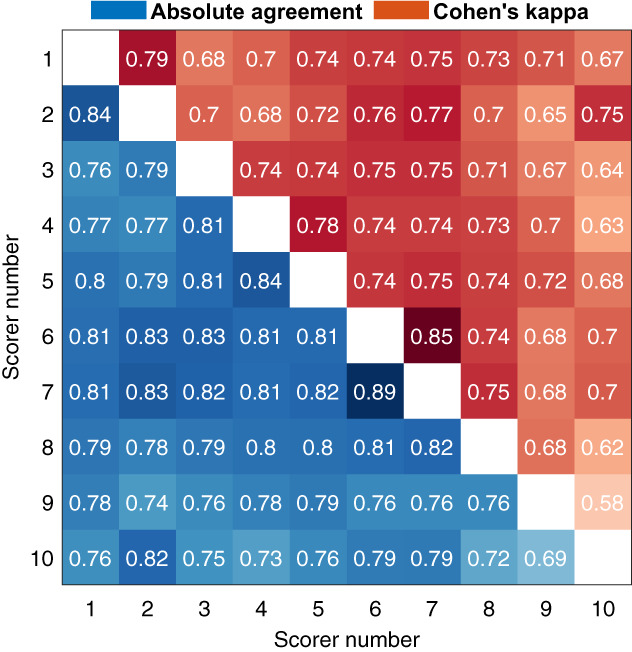
Pairwise agreement between the scorers. Absolute agreement is shown in the lower left and Cohen's kappa on the upper right.

**FIGURE 2:**
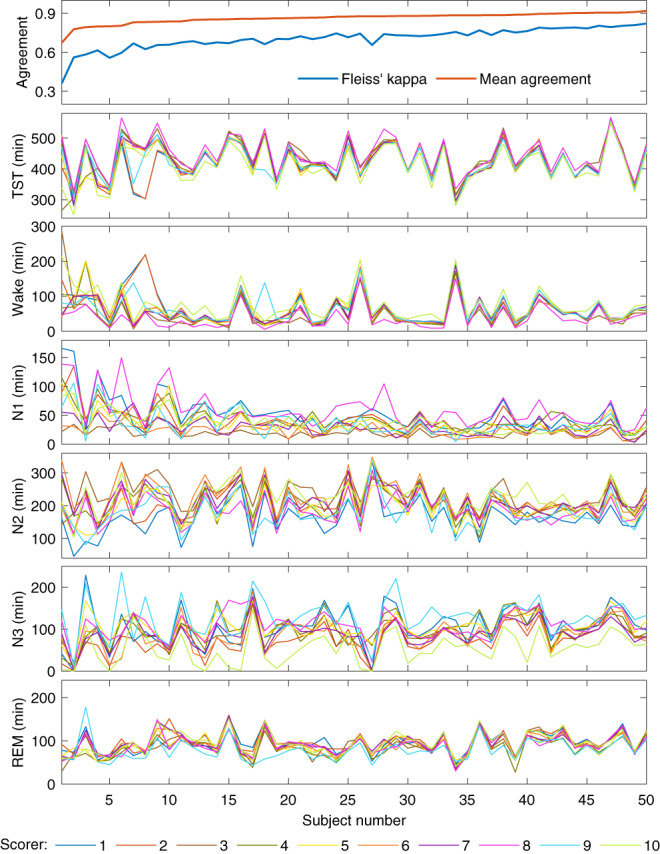
The specific scoring agreement for each of the 50 subjects and the amount of each sleep stage scored by all 10 scorers. The subjects are ordered from lowest to highest mean agreement. Mean agreement is calculated as the mean agreement with majority scoring.

**FIGURE 3:**
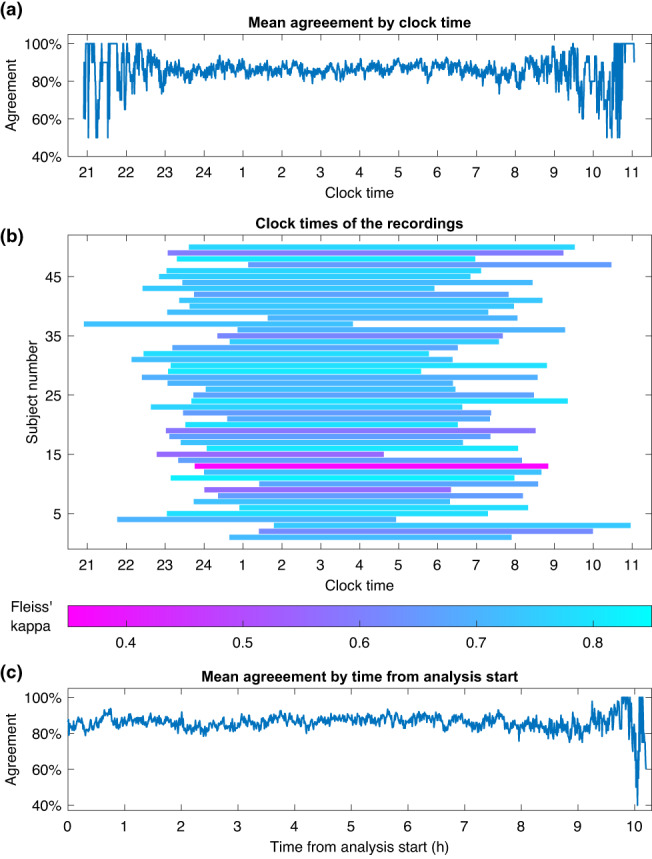
Mean agreement with majority score across all subjects during a specific clock time (a), the clock times when each recording was conducted (b), and the agreement as a function of time from analysis start (c).

The scorers were also compared pairwise (Figure 1). The lowest agreement was between scorers 9 and 10 at *κ*_c_ = 0.58. Scorers 9 and 10 also had the lowest overall agreement with all other scorers. Scorers 1 and 2, 4 and 5, and 6 and 7 were from the same sleep centres and also had the three highest pairwise *κ* values at 0.79, 0.85, and 0.78 respectively. Scorers 6 and 7 also reached the highest overall agreement with majority scoring. The scoring and scoring agreement also varied between the 50 subjects (Figure 2). Similarly, least variation was found in REM and the most variation was found in N1 also at the subject‐by‐subject level. Although there were considerable variations in the recordings lengths and clock times between the subjects, they had little effect on the scoring agreement (Figure 3).

**FIGURE 4:**
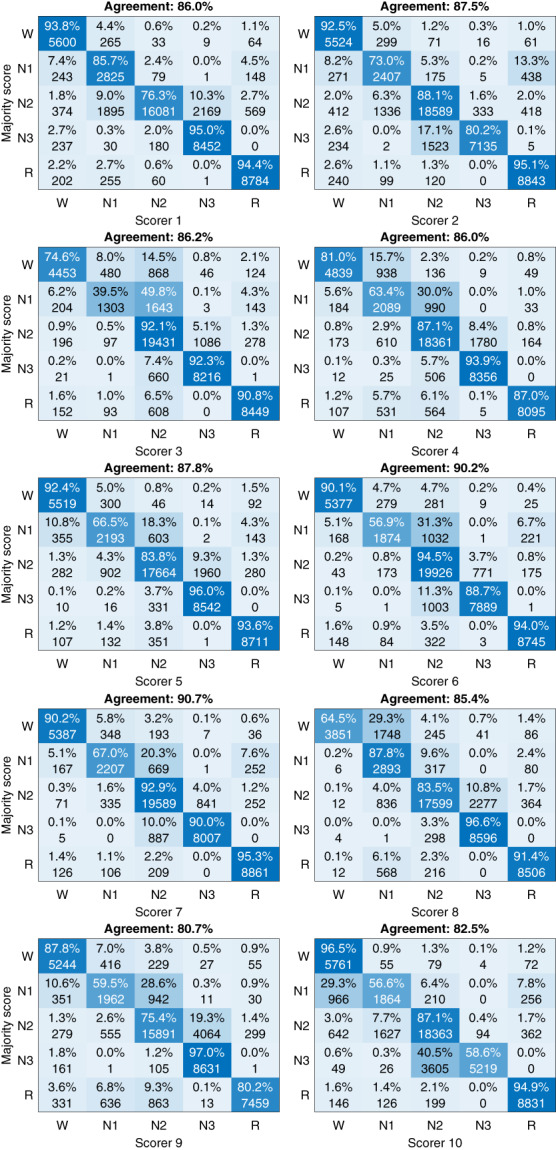
Confusion matrices against the majority score for each of the 10 scorers.

Confusion matrices against the majority score highlighted the differences between the scorers in scoring specific sleep stages (Figure 4). There was great variation in which sleep stage the disagreement occurred with no clear pattern between the scorers. For example, although scorer 9 had the lowest overall agreement with the majority score, the N3 agreement with the majority score was the highest. In addition, scorer 8 had a high overall agreement but had considerably lower wake agreement compared to the other scorers.

**FIGURE 5:**
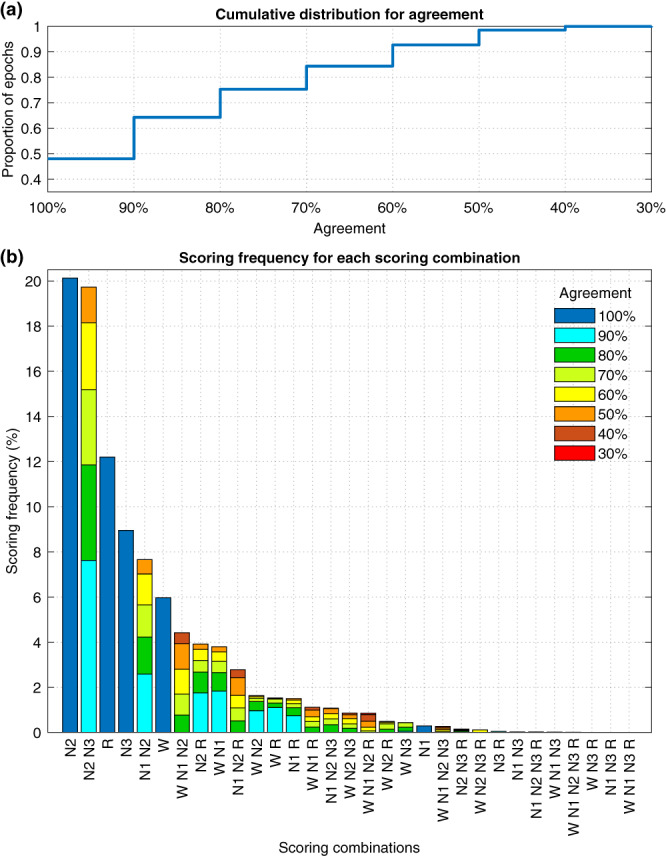
Cumulative distribution for the scoring agreement (a) and a bar chart of all scoring combinations and their proportion of all scored epochs from all 50 subjects (b).

**FIGURE 6:**
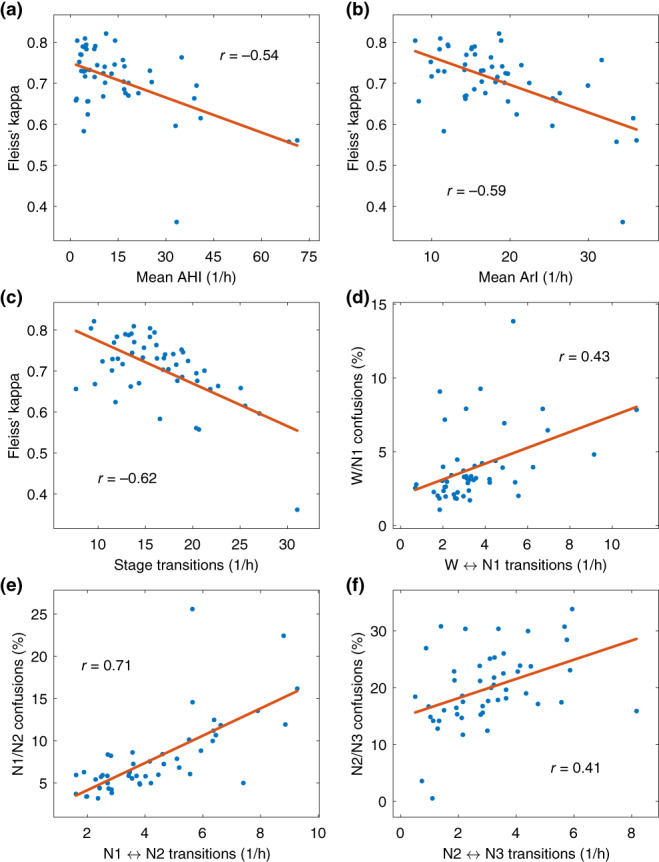
Scatter plots and correlation coefficients of mean apnea–hypopnea index (AHI) and Fleiss’ kappa (a), arousal index (ArI) and Fleiss’ kappa (b), rate of sleep stage transitions and Fleiss’ kappa (c), rate of wake (W)⟷N1 transitions and wake/N1 confusions (d), rate of N1⟷N2 transitions and N1/N2 confusions (e), and rate of N2⟷N3 transitions and N2/N3 confusions (f), for all 50 subjects. Transitions are expressed as transitions per hour and confusions as a percentage of the total analysed time. The red line shows a first‐degree linear least‐squares fit on the data.

Investigating the frequency and distribution of different scoring combinations showed that 48.0% of all scored stages reached 100% agreement among the scorers (Figure 5a). The most scored scoring combination was N2 with 100% agreement followed by N2/N3 with 50%–90% agreement (Figure 5b). All stages where the agreement was 100% were in the top six most scored combinations except for N1, which was only scored with 100% agreement in a total of 141 epochs (0.3%). Mean apnea–hypopnea index (AHI; Figure 6a) arousal index (ArI; Figure 6b) and the frequency of stage transitions (Figure 6c) were negatively correlated with the scoring agreement. The frequency of stage transitions was also correlated with the proportion of confusions between the stages for wake/N1 (Figure 6d), N1/N2 (Figure 6e), and N2/N3 (Figure 6f).

## 4 DISCUSSION

Although the overall agreement between the scorers was high with *κ*_f_ = 0.714 and mean agreement with the majority of 0.863, there was considerable variation in the agreement between the sleep stages (Table 3). The agreements presented in the present study are mostly in line with previous studies, which have reported an overall agreement of between *κ* = 0.57 and *κ* = 0.76 (Danker‐Hopfe et al., 2009; Magalang et al., 2013; Norman et al., 2000; Penzel et al., 2013; Rosenberg & Van Hout, 2013; Zhang et al., 2015). More detailed agreements reported in previous studies are presented in Table 5. The relatively lower wake agreement in the present study is likely due to the fact that all excess wake was trimmed from the start and end of the recordings. The wake periods before the subject first falls asleep and the periods after they wake up in the morning should be easier to score and including large portions of this type of wake would over‐inflate the wake accuracy. It should be also noted that the agreement values are not directly comparable between the studies as the values are not calculated the same exact way due to the differing study setups, subject populations, number of recordings, and number of scorers. In addition, how the sleep stage‐specific agreements and *κ* values were determined, have not always been fully elaborated (Danker‐Hopfe et al., 2009; Magalang et al., 2013; Rosenberg & Van Hout, 2013; Zhang et al., 2015).

**TABLE 5:** Sleep stage agreement reported in previous studies.

Sleep stage	Present study	Magalang et al. (2013)	Zhang et al. (2015)	Rosenberg & Van Hout (2013)	Danker‐Hopfe et al. (2009)
All stages	*κ*_f_ = 0.71	*κ* = 0.63	*κ* = 0.57	83%	*κ* = 0.86
Wake	*κ*_fb_ = 0.76	*κ* = 0.78	*κ* = 0.65	84%	*κ* = 0.86
N1	*κ*_fb_ = 0.41	*κ* = 0.31	*κ* = 0.16	63%	*κ* = 0.46
N2	*κ*_fb_ = 0.67	*κ* = 0.60	*κ* = 0.58	85%	*κ* = 0.72
N3	*κ*_fb_ = 0.74	*κ* = 0.67	*κ* = 0.49	67%	*κ* = 0.73
REM	*κ*_fb_ = 0.86	*κ* = 0.78	*κ* = 0.79	90%	*κ* = 0.91

Abbreviations: *κ*_f_, Fleiss’ kappa; REM, rapid eye movement.

The agreement was highest when the majority sleep stage was REM. N3 had also a higher‐than‐average agreement while N1 had the lowest agreement. The low agreement in N1 was expected as it has also been low in previous studies (Danker‐Hopfe et al., 2009; Magalang et al., 2013; Rosenberg & Van Hout, 2013; Zhang et al., 2015). Thus, we also evaluated the agreement of N1 and N2 combined into a light sleep stage. This is often done in, e.g., automatic sleep staging applications as it has been noticed that many models have great difficulty separating N1 and N2 correctly (Korkalainen et al., 2019, 2020). When combined, the mean agreement with majority scoring was 0.827, while with standard five‐stage scoring, it was 0.656 for N1 and 0.860 for N2. The agreement values also slightly increased for all other stages, which was to be expected as the total number of classes was reduced by one.

From the agreement values, it is not fully clear whether combining N1 and N2 actually significantly increases the agreement or whether the much larger amount of N2 (Table 4) simply dominates the light sleep stage, and thus, the agreement appears higher. In addition, there is considerable disagreement between wake and N1 (Figures 4 and 5b) that the change to light sleep stage would not affect. However, the correlation between confusions and transitions was considerably higher in N1/N2 than in wake/N1 or N2/N3 (Figure 6). In addition, mixed N1‐N2 was the fifth most scored scoring combination with a 7.7% frequency, while N1 alone was only scored with a 0.3% frequency (Figure 5b).

Furthermore, while overall 48.0% of all scored epochs reached 100% agreement within the scorers (Figure 5a), in N1 this was managed in only 141 total epochs, which is 4.3% of the N1 epochs defined by the majority scoring. In contrast, all the other sleep stages where the agreement was 100% were in the top six most scored combinations with frequency around half the total frequency of the stage (Figure 5b, Table 4). Thus, there is a clear indication that differentiating between N1 and N2 is difficult and that there is almost never full confidence in N1 scoring, even for expert sleep technologists using the same scoring rules.

**FIGURE 7:**
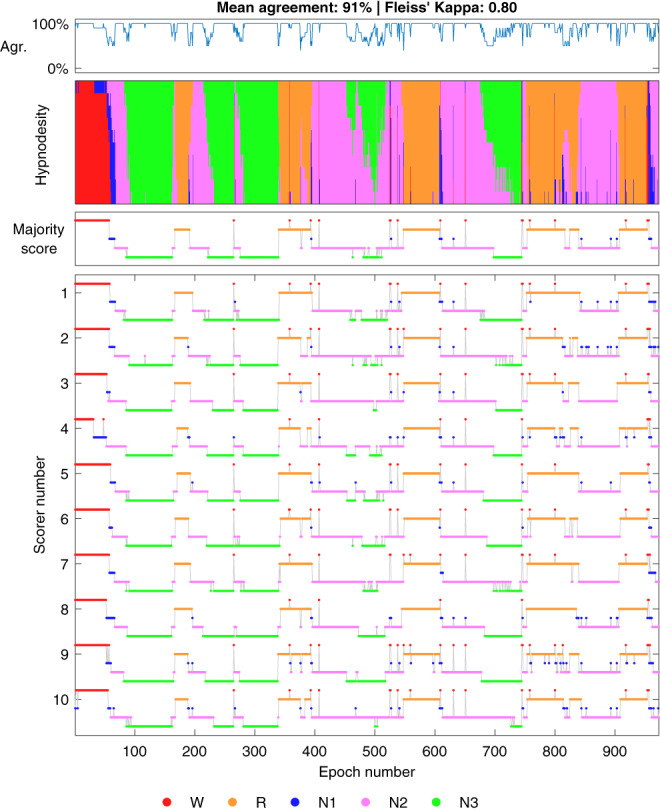
Example of a subject with a high inter‐scorer agreement. Hypnodensity represents a stacked histogram of scored stages for each epoch. Agr., agreement with majority score; R, rapid eye movement; W, wake.

**FIGURE 8:**
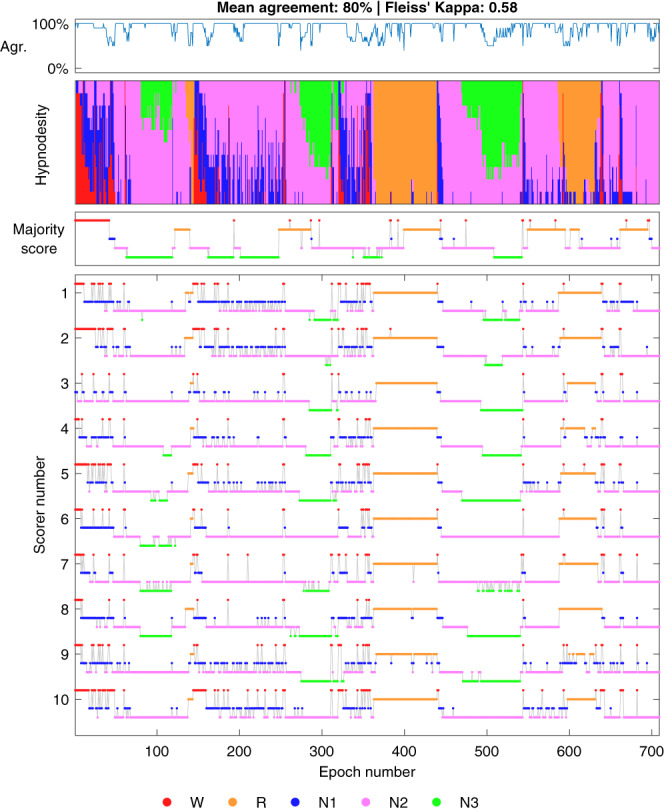
Example of a subject with a low inter‐scorer agreement. Hypnodensity represents a stacked histogram of scored stages for each epoch. Agr., agreement with majority score; R, rapid eye movement; W, wake.

One of our hypotheses was that stage transitions would be areas of low agreement as it might be difficult to judge accurately the exact point when the transition occurs. This was supported by our analyses as we found that there was a negative correlation between the number of stage transitions and the sleep staging agreement (Figure 6c). This effect is also evident from Figures 7 and 8, where the agreement consistently drops during stage transitions while long periods of the same sleep stage usually eventually reach a perfect agreement with all scorers. One likely reason for the drop in agreement during the transitions is that often epochs start as one stage and end as another, e.g., N2–N3 transition. Thus, even if all scorers would agree that a transition happens during the same epoch, some scorers may still consider it to be more N2 while some would score it N3. Thus, it is expected that the agreement drops at least briefly during stage transitions. The low agreement during stage transitions could also at least partially explain the low agreement in N1 as the length of N1 cycles is usually quite short and thus, the stage transition areas are therefore a much larger portion of the total amount of N1. The number of respiratory events and arousals were similarly correlated to the scoring agreement as the stage transitions (Figures 6a–c). This is also not surprising as many of these stage transitions are arousals, which are in turn caused by respiratory events. Thus, these effects are not independent.

However, the stage transitions are certainly not the only or even necessarily the predominant reason for disagreement in the scoring as the wake/N1 and N2/N3 confusions percentages showed only weak correlations with wake⟷N1 and N2⟷N3 transitions (Figures 6d,f). Although the correlation was considerably higher between N1/N2 transitions and confusions (Figure 6e). Considering all of the agreement metrics, neither the low amount of N1 nor the stage transitions can fully explain the low agreement in N1, and it inherently seems to be considerably more difficult to score. One possible explanation for this difficulty could be the individual differences in sleep onset alpha activity, as alpha frequencies vary markedly between individuals and ~10% of the general population display no alpha rhythm upon eye closure (Berry et al., 2020).

Interestingly, there were large variations where each scorer disagreed with the majority score (Figure 4). For example, scorer 1 reached a very high agreement in N1 (85.7%) but the N2 agreement was one of the lowest (76.3%). Similarly, scorer 8 had a very high agreement in all other sleep stages, but the wake agreement was by far the lowest (64.5%). Scorer 10 only reached 58.6% agreement in N3 where there was an otherwise high agreement between all other scorers. REM was the only stage where each scorer reached ≥80% agreement with the majority score and it was also the stage with the highest agreement in all metrics. It is good to note, that as the largest differences were between the NREM stages, the disagreement may be less crucial from a clinical perspective as most OSA metrics only consider the total sleep time and a NREM/REM distinction to assess REM‐related OSA. This can also be seen in Figure 2, where although there is considerable variation between the scorers, the scorers mostly follow the same trend between subjects, and the total sleep time is not affected as much. However, there were also differences in sleep/wake scoring as, e.g., scorer 8 scored considerably less wake than other scorers (Table 4, Figure 4). Overall, these findings show that the disagreement in scoring is not only due to events or stage transitions but that there seem to be significantly different interpretations of the same visual‐scoring rules. For example, in N3 scoring, some disagreement might be caused by a different propensity to count the exact length of all delta waves within a given epoch versus looking at the overall morphology of the epoch without exact measurements. Differences in arousal scoring might also have an effect as some scorers may score arousals where there are delta waves with superimposed alpha activity and exclude these segments when counting the length of delta activity in the epochs. Being from the same sleep centre reduced the variation between the scorers as expected (Figure 1), although there were still considerable differences.

The overall agreement remained mostly the same regardless of the clock time and there was no apparent improvement or decrease in scoring agreement during the night (Figure 3a). More variation is visible in the early and late hours. However, this is simply because fewer subjects are sleeping during those hours. In comparison, during the middle of the night, the mean agreement is calculated from all 50 subjects, which limits the variation. The start or end time, or the length of the recording had no apparent effect on the scoring agreement either (Figure 3b). No apparent change in the agreement was found from the beginning of the analysis to the end of the analysis either (Figure 3c). This indicates that there was no significant loss of attention, or familiarisation to the specific EEG features of the subject during the night.

This study also has certain limitations. The tiebreaker method used when forming the majority score may have a slight impact on the results as 3.6% of the epochs resulted in a tie between two or more stages. However, this should only be a minimal effect as in these cases the agreement must already be very low that is ≤50%. The areas with low agreement would also still stay the same and, e.g., the mean agreement with majority scoring would be exactly the same regardless of the tiebreaker method. Some recordings had issues with signal quality and sensor connections, and some scorers handled these segments differently from others. For example, if the pulse oximeter was disconnected for a part of the recording, most scorers still scored sleep stages as pulse oximetry is not required for sleep staging, while some scorers marked these segments as invalid periods or artefacts and thus did not perform sleep staging. For those scorers who did not score these sections, these epochs were considered as wake in the agreement analyses. We chose not to include an additional ‘unscored’ stage as only 0.2% of all scored epochs were labelled as unscored. Instead, we considered these epochs as wake as it is the closest stage for these epochs as artefacts and invalid periods are not counted towards total sleep time and no respiratory events would be scored over them either. Finally, as the recordings were type II PSGs, they included varying amounts of wake before and after the actual sleep period, ranging from a few minutes to multiple hours, with no reliable markings for ‘lights‐on’ or ‘lights‐off’ times. This makes accurately determining sleep latency and sleep efficiency difficult. Therefore, as there were no reliable markings that could be used to determine the time in bed, we elected to trim all excess wake periods from the start and the end, even though the sleep latency period may be partially cut also. We chose this approach over including an arbitrary amount of wake before and after the sleep period as this would have only caused a positive bias to the accuracy of wake scoring.

In conclusion, although the overall sleep staging agreement was high, there are several areas for improvement. The most frequent disagreement was in the NREM stages, especially in N1 sleep. As there is almost never 100% agreement in N1 scoring, there may be a need to re‐evaluate its value in sleep staging and whether it should be scored separately in the future. In addition, although stage transitions were identified as a partial cause of disagreement, there seem to be fundamental differences in how different scorers perform sleep staging. As the agreement was higher between the scorers from the same sleep centres, the disagreement is likely at least partially due to different interpretations of the same scoring rules. Thus, it may be necessary to re‐evaluate and improve some of the scoring rules if the sleep staging agreement is to be improved.

## AUTHOR CONTRIBUTIONS

**Sami Nikkonen:** Conceptualization; data curation; formal analysis; funding acquisition; investigation; methodology; software; visualization; writing – original draft; writing – review and editing. **Pranavan Somaskandhan:** Methodology; visualization; writing – review and editing. **Henri Korkalainen:** Methodology; writing – review and editing. **Samu Kainulainen:** Writing – review and editing. **Phil Terrill:** Writing – review and editing. **Heidur Gretarsdottir:** Data curation; writing – review and editing. **Sigridur Sigurdardottir:** Data curation; writing – review and editing. **Kristin Anna Olafsdottir:** Project administration. **Anna Sigridur Islind:** Writing – review and editing. **Maria Oskarsdottir:** Writing – review and editing. **Erna S. Arnardóttir:** Funding acquisition; project administration; writing – review and editing. **Timo Leppänen:** Conceptualization; funding acquisition; project administration; writing – review and editing.

## CONFLICT OF INTEREST STATEMENT

The authors declare no conflicts of interest.

## Data Availability

Research data are not shared.

## References

[jsr13956-bib-0001] Arnardottir, E. S. , Islind, A. S. , Óskarsdóttir, M. , Ólafsdóttir, K. A. , August, E. , Jónasdóttir, L. , Hrubos‐Strøm, H. , Saavedra, J. M. , Grote, L. , Hedner, J. , Höskuldsson, S. , Ágústsson, J. S. , Jóhannsdóttir, K. R. , McNicholas, W. T. , Pevernagie, D. , Sund, R. , Töyräs, J. , & Leppänen, T. (2022). The sleep revolution project: The concept and objectives. Journal of Sleep Research, 31(4), e13630. 10.1111/JSR.13630 35770626

[jsr13956-bib-0002] Bakker, J. P. , Ross, M. , Cerny, A. , Vasko, R. , Shaw, E. , Kuna, S. , Magalang, U. J. , Punjabi, N. M. , & Anderer, P. (2022). Scoring sleep with artificial intelligence enables quantification of sleep stage ambiguity: Hypnodensity based on multiple expert scorers and auto‐scoring. Sleep, 46(2). 10.1093/SLEEP/ZSAC154 PMC9905781 35780449

[jsr13956-bib-0003] Benediktsdottir, B. , Janson, C. , Lindberg, E. , Arnardóttir, E. S. , Olafsson, I. , Cook, E. , Thorarinsdottir, E. H. , & Gislason, T. (2010). Prevalence of restless legs syndrome among adults in Iceland and Sweden: Lung function, comorbidity, ferritin, biomarkers and quality of life. Sleep Medicine, 11(10), 1043–1048. 10.1016/j.sleep.2010.08.006 20961808

[jsr13956-bib-0004] Berry, R. B. , Brooks, R. , Gamaldo, C. E. , Harding, S. M. , Lloyd, R. M. , Quan, S. F. , Troester, M. M. , Vaughn, B. V. , & For the American Academy of Sleep Medicine . (2020). AASM manual for the scoring of sleep and associated events: Rules, terminology and technical specifications (2.6). American Academy of Sleep Medicine.

[jsr13956-bib-0005] Chung, F. , Abdullah, H. R. , & Liao, P. (2016). STOP‐bang questionnaire: A practical approach to screen for obstructive sleep apnea. Chest, 149(3), 631–638. 10.1378/CHEST.15-0903 26378880

[jsr13956-bib-0006] Cohen, J. (1960). A coefficient of agreement for nominal scales. Educational and Psychological Measurement, 20(1), 37–46.

[jsr13956-bib-0007] Danker‐Hopfe, H. , Anderer, P. , Zeitlhofer, J. , Boeck, M. , Dorn, H. , Gruber, G. , Heller, E. , Loretz, E. , Moser, D. , Parapatics, S. , Saletu, B. , Schmidt, A. , & Dorffner, G. (2009). Interrater reliability for sleep scoring according to the Rechtschaffen & Kales and the new AASM standard. Journal of Sleep Research, 18(1), 74–84. 10.1111/j.1365-2869.2008.00700.x 19250176

[jsr13956-bib-0008] Fleiss, J. L. (1971). Measuring nominal scale agreement among many raters. Psychological Bulletin, 76(5), 378–382. 10.1037/H0031619

[jsr13956-bib-0009] Horiguchi, J. , Hornyak, M. , Voderholzer, U. , Kryger, M. , Skomrow, R. , Lipinski, J. F. , Masood, A. , Phillips, B. , Oertel, W. H. , Stiasny, K. , O'Keeffe, S. , Oldani, A. , Zucconi, M. , Ondo, W. G. , Picchietti, D. , Poceta, J. S. , Rich, G. B. , Scrima, L. , Shafor, R. , … Walters, A. S. (2003). Validation of the international restless legs syndrome study group rating scale for restless legs syndrome. Sleep Medicine, 4(2), 121–132. 10.1016/S1389-9457(02)00258-7 14592342

[jsr13956-bib-0010] Korkalainen, H. , Aakko, J. , Duce, B. , Kainulainen, S. , Leino, A. , Nikkonen, S. , Afara, I. O. , Myllymaa, S. , Töyräs, J. , & Leppänen, T. (2020). Deep learning enables sleep staging from photoplethysmogram for patients with suspected sleep apnea. Sleep, 43, 1–10. 10.1093/sleep/zsaa098 PMC7658638 32436942

[jsr13956-bib-0011] Korkalainen, H. , Leppanen, T. , Aakko, J. , Nikkonen, S. , Kainulainen, S. , Leino, A. , Duce, B. , Afara, I. O. , Myllymaa, S. , & Toyras, J. (2019). Accurate deep learning‐based sleep staging in a clinical population with suspected obstructive sleep apnea. IEEE Journal of Biomedical and Health Informatics, 5133, 2073–2081. 10.1109/jbhi.2019.2951346 31869808

[jsr13956-bib-0012] Magalang, U. J. , Chen, N. H. , Cistulli, P. A. , Fedson, A. C. , Gíslason, T. , Hillman, D. , Penzel, T. , Tamisier, R. , Tufik, S. , Phillips, G. , & Pack, A. I. (2013). Agreement in the scoring of respiratory events and sleep among international sleep centers. Sleep, 36(4), 591–596. 10.5665/sleep.2552 23565005 PMC3612261

[jsr13956-bib-0013] Morin, C. M. , Belleville, G. , Bélanger, L. , & Ivers, H. (2011). The insomnia severity index: Psychometric indicators to detect insomnia cases and evaluate treatment response. Sleep, 34(5), 601–608. 10.1093/SLEEP/34.5.601 21532953 PMC3079939

[jsr13956-bib-0014] Nikkonen, S. , Korkalainen, H. , Töyräs, J. , & Leppänen, T. (2022a). STAR sleep recording export software. Zenodo. 10.5281/zenodo.6139577 PMC9508159 36151239

[jsr13956-bib-0015] Nikkonen, S. , Korkalainen, H. , Töyräs, J. , & Leppänen, T. (2022b). STAR sleep recording export software for automatic export and anonymization of sleep studies. Scientific Reports, 12(1), 1–7. 10.1038/s41598-022-19892-0 36151239 PMC9508159

[jsr13956-bib-0016] Norman, R. G. , Pal, I. , Stewart, C. , Walsleben, J. A. , & Rapoport, D. M. (2000). Interobserver agreement among sleep scorers from different centers in a large dataset. Sleep, 23(7), 901–908. 10.1093/sleep/23.7.1e 11083599

[jsr13956-bib-0017] Penzel, T. , Zhang, X. , & Fietze, I. (2013). Inter‐scorer reliability between sleep centers can teach us what to improve in the scoring rules. Journal of Clinical Sleep Medicine, 9(1), 89–91. 10.5664/jcsm.2352 23319911 PMC3525995

[jsr13956-bib-0018] Rechtschaffen, A. , & Kales, A. (1968). A manual of standardized terminology, techniques and scoring system for sleep stages in human subjects. Brain Information Service. 10.1046/j.1440-1819.2001.00810.x 11422885

[jsr13956-bib-0019] Rosenberg, R. S. , & Van Hout, S. (2013). The American academy of sleep medicine inter‐scorer reliability program: Sleep stage scoring. Journal of Clinical Sleep Medicine, 9(1), 81–87. 10.5664/jcsm.2350 23319910 PMC3525994

[jsr13956-bib-0020] Younes, M. , Raneri, J. , & Hanly, P. (2016). Staging sleep in Polysomnograms: Analysis of inter‐scorer variability. Journal of Clinical Sleep Medicine, 12(6), 885–894. 10.5664/JCSM.5894 27070243 PMC4877322

[jsr13956-bib-0021] Zhang, X. , Dong, X. , Kantelhardt, J. W. , Li, J. , Zhao, L. , Garcia, C. , Glos, M. , Penzel, T. , & Han, F. (2015). Process and outcome for international reliability in sleep scoring. Sleep and Breathing, 19(1), 191–195. 10.1007/s11325-014-0990-0 24801137

